# Implementation and Evaluation of Walk-in-Place Using a Low-Cost Motion-Capture Device for Virtual Reality Applications

**DOI:** 10.3390/s24092848

**Published:** 2024-04-30

**Authors:** Rawoo Shin, Bogyu Choi, Sang-Min Choi, Suwon Lee

**Affiliations:** Department of Computer Science, Gyeongsang National University, Jinju-si 52828, Republic of Korea; rawoo91@gnu.ac.kr (R.S.); chlqhrb8769@naver.com (B.C.)

**Keywords:** virtual reality, motion capture, walk-in-place, locomotion, absolute trajectory error, head-mounted display

## Abstract

Virtual reality (VR) is used in many fields, including entertainment, education, training, and healthcare, because it allows users to experience challenging and dangerous situations that may be impossible in real life. Advances in head-mounted display technology have enhanced visual immersion, offering content that closely resembles reality. However, several factors can reduce VR immersion, particularly issues with the interactions in the virtual world, such as locomotion. Additionally, the development of locomotion technology is occurring at a moderate pace. Continuous research is being conducted using hardware such as treadmills, and motion tracking using depth cameras, but they are costly and space-intensive. This paper presents a walk-in-place (WIP) algorithm that uses Mocopi, a low-cost motion-capture device, to track user movements in real time. Additionally, its feasibility for VR applications was evaluated by comparing its performance with that of a treadmill using the absolute trajectory error metric and survey data collected from human participants. The proposed WIP algorithm with low-cost Mocopi exhibited performance similar to that of the high-cost treadmill, with significantly positive results for spatial awareness. This study is expected to contribute to solving the issue of spatial constraints when experiencing infinite virtual spaces.

## 1. Introduction

Virtual reality (VR) is a technological revolution that combines the real and virtual worlds to transport users to various novel environments. It can present environments and situations that may be difficult to experience in real life and offers innovative experiences in entertainment, education, training, medicine, and many other fields [1]. VR technology integrates visual, auditory, and physical senses to provide users with an experience that closely resembles reality, and various technical tools are essential for enabling interactions that mimic real-world behavior within the VR environment. Currently, accurately reflecting real-world movements in VR is challenging due to spatial and technical constraints associated with recognizing movements in real environments and reflecting them in virtual ones [2]. Although large virtual spaces can be theoretically implemented in VR, spatial constraints are encountered when implementing movements such as walking. These include tracking hardware and network limitations, physical obstacles, and intricate technical issues related to conveying user movements to the virtual environment [3].

A potential solution to these challenges is to improve locomotion in VR environments [4]. Locomotion refers to controlling user movements within the virtual environment and is a crucial factor for improving user experience [5]. Methods that enable users to move naturally in VR environments as they would while walking or running during everyday activities in the real world are being actively researched. Locomotion research has employed various methods, with walk-in-place (WIP) being the most actively researched [2,6]. An evaluation of locomotion methods demonstrated that users with no prior VR experience rated WIP as being more immersive than controller manipulation [5]. Various methods have been proposed for implementing WIP, wherein the Kinect camera, which can measure depth [7,8], trackers that can track body movements [9], treadmills [10], or body-worn inertial measurement unit sensors have been employed [11].

Developing WIP techniques that can represent real-world movements in VR environments has been a long-standing challenge. Despite numerous technological advancements, several gaps remain, primarily the limited amount of physical space. The main objective of WIP techniques is to provide an immersive experience by translating real-world movements into the virtual world and simulating an infinite virtual space within a confined physical space. However, WIP experiences through previously proposed methods require considerable space to set up the camera and perform actions in a confined space or using bulky hardware such as a treadmill. Additionally, establishing a WIP environment is expensive, and accurate tracking technology is required to replicate real-world motion in VR environments. However, procuring multiple Kinect cameras, trackers, sensors, and treadmills can entail significant costs.

This paper presents a WIP algorithm that uses low-cost motion-capture equipment and demonstrates its potential for use in VR applications. By monitoring the walking behavior in real time using body-worn sensors and applying the collected data to the WIP algorithm, the system can enhance user immersion by providing meaningful interactions in virtual worlds. In the performance evaluations, the absolute trajectory errors (ATEs) were compared with the 3D coordinates obtained by walking along a specified path in VR using Mocopi and a treadmill. The final performance was evaluated based on the results of a user survey.

The remainder of this paper is organized as follows. Section 2 presents a detailed review of related studies. Section 3 describes the proposed configuration and algorithm for WIP implementation. Section 4 analyzes and discusses the experimental results to evaluate the performance of the proposed technique. Section 5 discusses the limitations, and theoretical and managerial implications, of the study. Section 6 summarizes the significance and findings of the study and presents the conclusions.

## 2. Related Works

VR is a revolutionary technology that allows users to interact with the real world by transporting them into virtual environments that differ from the real world. However, replicating natural movements in these environments remains technically complex. WIP technology is a key solution for mimicking the walking behavior of a user in a VR environment, and considerable research and development has been conducted in this field. A key aspect of WIP research is the development and exploration of various techniques, which has contributed to improving both the user experience and system performance.

For instance, the use of hardware such as bracelets to track arm movements while walking has been explored. The obtained data can be interpreted as movement commands using algorithms that enable movement within a virtual space [12]. Implementing WIP using hands can be simple and offer high accuracy. However, this implies the standard hand-based interaction typically used for interaction in the virtual world becomes unfeasible because the hands are employed for this specific purpose.

Another area of research involves techniques that utilize the leg movements that accompany regular movements. These WIP methods leverage the physical characteristics of how humans lift their legs while walking to track the motion of the lifted leg. Thus, they imitate real-world walking in more detail, and various types of WIP methods are being developed. Initially, a complex mix of sensors was used, but advances in small-scale sensor technology have enabled the utilization of sensors built into head-mounted displays (HMDs), smartphones, and Kinect cameras. These methods can track body movements in detail and provide users with realistic walking experiences.

WIP techniques that employ Kinect cameras are divided into single- and multi-camera methods. Single-camera methods feature high body recognition rates when the user is looking straight ahead or turning; however, they face issues in tracking occluded areas when the body is turned at an angle or sideways. Therefore, multicamera methods have been widely studied to address the limitations of single cameras [13,14,15].

However, implementing WIP using a Kinect camera presents the challenge of moving within the camera’s field of view owing to its distance and space limitations [16]. Methods that employ physical sensors offer a more robust tracking performance; however, depending on the sensor, they may require external assistance or incur significant costs. Additionally, various WIP techniques have been studied, including tracking and extracting data from physical motion or mechanically determining the walking motion [2]. Therefore, WIP research is ongoing with the aim of understanding the diversity of WIP technologies, their strengths and weaknesses, and ways to improve them [9].

Researchers have also investigated the use of tracking devices attached to the body, such as the VIVE tracker, which exhibits high accuracy [17]. However, these trackers re-quire external sensors owing to their inherent hardware characteristics. Body-attached tracking devices may miss tracks if the user moves out of the sensor area or the tracker is obstructed by an object. Additionally, compatibility issues may arise when using devices other than the specified device depending on the type of tracker used. Moreover, research on using treadmills for WIP applications is ongoing with constant hardware advancements [18]. This method offers the advantage of virtually replicating the real-world movements of users. However, the cost of creating such an environment remains challenging.

Recent research on WIP has focused on 360-degree omnidirectional walking and treadmills as important locomotion technologies, along with omnidirectional walking trackers attached to the body to mimic larger-than-life environments [19]. These methods offer locomotion without the need for a large physical space and induce lower dizziness than traditional locomotion techniques [20]. However, they are expensive or bulky, making their commercialization challenging.

These findings indicate that WIP is an important technique for mimicking locomotion in VR environments and various studies continue to improve its performance and evaluate its advantages and disadvantages. This continued effort to provide a natural VR experience plays a significant role in the development of VR technologies.

## 3. Materials and Methods

This section describes the setup employed for detecting user movements and implementing the proposed WIP technique. Real-time body data obtained through motion-capture technologies are necessary to ensure smooth virtual interactions. Various devices exist for obtaining accurate body data, such as Kinect cameras, which capture the body from the outside, or sensors attached to the body. Therefore, this chapter introduces the hardware selection and communication methods, along with algorithms for recognizing body data collected via motion-capture devices and implementing WIP.

### 3.1. Trackers

Various types of hardware at different price points are available that involve attaching sensors to the body to collect data, including acceleration, gyro, and pressure sensors. This study employed the Mocopi system comprising six sensors, each weighing approximately 8 g, owing to its ease of use and accurate tracking performance. These sensors must be attached to the head, waist, wrist, and ankle for tracking. Additionally, Mocopi is 20 times cheaper than a treadmill and 2.5 times cheaper than the VIVE Sensor and, offers excellent tracking performance.

### 3.2. HMDs

An HMD displays a virtual world in 3D through two displays situated in front of the eyes, thereby creating a realistic and immersive experience. This study focused on Meta Quest 2 and VIVE Pro HMDs as visual aids for VR; however, other HMDs have also been developed for this purpose. This paper reports on the experiments conducted using Meta Quest 2 and VIVE Pro for implementing the proposed WIP algorithm using Mocopi and a treadmill, respectively.

### 3.3. Treadmill

A treadmill can be used to implement the walking or running motion of a user in a VR environment. It provides a realistic experience by allowing users to physically move around a virtual world. However, it has some disadvantages such as high cost and weight, bulkiness, and space limitations for installation. However, we selected it as a comparison device for implementing the proposed WIP algorithm owing to its high tracking accuracy and immersiveness compared to other locomotion hardware. Additionally, its performance was compared with that of Mocopi using ATEs and user surveys.

### 3.4. Data Communication between Smartphone and PC

Next, the process of sending and receiving data through Mocopi is described. There is a direct connection between Mocopi and a PC, but not in real time, so we used Bluetooth. First, we ran the Mocopi application on a smartphone and established a Bluetooth connection between the smartphone and Mocopi. The data were then transferred via Wi-Fi to a PC on the same network for visualization. The communication and internet protocols used were the user datagram protocol and IPv4, respectively.

### 3.5. Data Communication between PC and HMD

To enable 360-degree WIP, the rotation of the user must be unrestricted. This study aimed to enhance freedom of movement without using a data cable, which limits rotation. When using Meta Quest 2, the screen data are wirelessly transferred from the PC to the HMD through the air-link function, which is available when the PC and HMD are on the same network and uses the 5 GHz channel to prevent communication delays that may cause cognitive dissonance in the user. VIVE Pro allows unrestricted user movement owing to the absence of wires connecting the HMD to the PC and, instead, employs a special cable attached to the ceiling. Additionally, it is connected to a PC via a DisplayPort cable, and visual data are sent and received through a hub.

### 3.6. Calibration

Prior to motion capture, the equipment must be calibrated to obtain accurate body data. Mocopi uses a dedicated application for calibration, which involves a straightforward process. However, errors can accumulate over time owing to the nature of the equipment, necessitating regular calibration. 

Prior to calibration, data pertaining to the position of the sensors and the user’s information are collected. This is based on three pieces of information: the user’s height, their default pose, and their movement from the default pose to the default pose once more. Based on the aforementioned data, the user’s pose error is calibrated. We performed calibration at the start of the study and after any change in location.

### 3.7. Motion Capture

Motion-capture technology extracts 3D data of the body movements using sensors or cameras, and we used calibrated Mocopi data to estimate the joint positions and postures of users. Mocopi detects the acceleration and angular velocity of body movements using six sensors that must be worn on the head, waist, wrists, and ankles to calculate and apply 3D positions and postures. It uses 3 accelerometers and 3 gyroscopes to digitize the movements for determining the sensor positions and acceleration data to identify the 3D position. Up to this point, positioning errors accumulate in the system and reduce accuracy, but Sony’s proprietary artificial intelligence (AI) model directly estimates the joint positions to minimize positioning errors [21,22,23]. 

**Figure 1:**
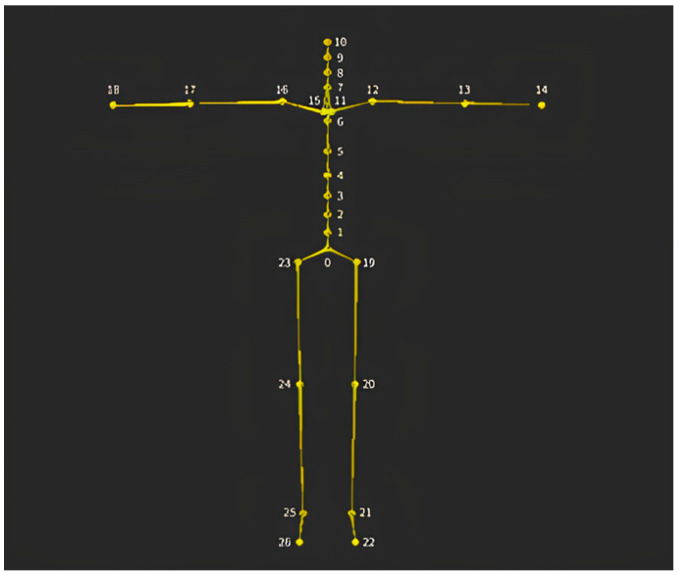
Skeletal structure defined in Mocopi. The numbers are the indices of the joints, and the names of the joints are organized in Table 1.

**Figure 2:**
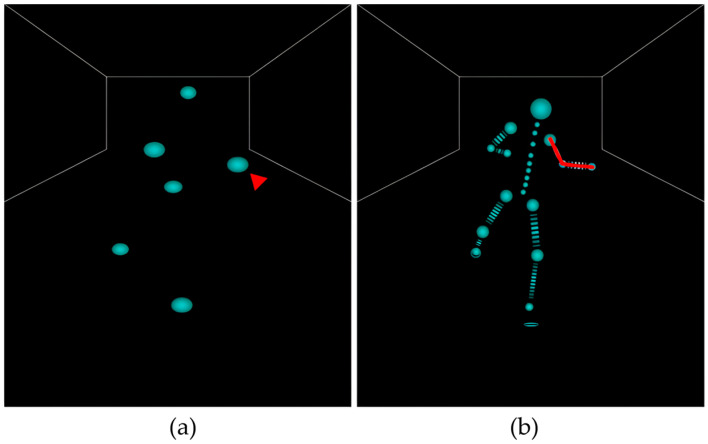
(**a**) Sensor-attached joint positions and (**b**) estimated joint positions where sensors are not attached. From the left wrist position marked in red in (**a**), the positions of the left shoulder and elbow, indicated by the red line in (**b**), are estimated.

The problem with tracking the positions of joints not attached to the sensors is that owing to the complexity of the human body and the high degrees of freedom of the joints, the position and posture of the intermediate joints connecting two specific locations where the sensors are attached cannot be uniquely determined through simple geometric calculations. However, the AI models trained on different human movements are calibrated to the natural positions of the joints. Figure 1 and Table 1 show the human skeletal structure defined by Mocopi, and Figure 2 shows the positions of the joints without sensors estimated from the locations of the sensor attachments.

### 3.8. WIP Algorithm with Mocopi

**Figure 3:**
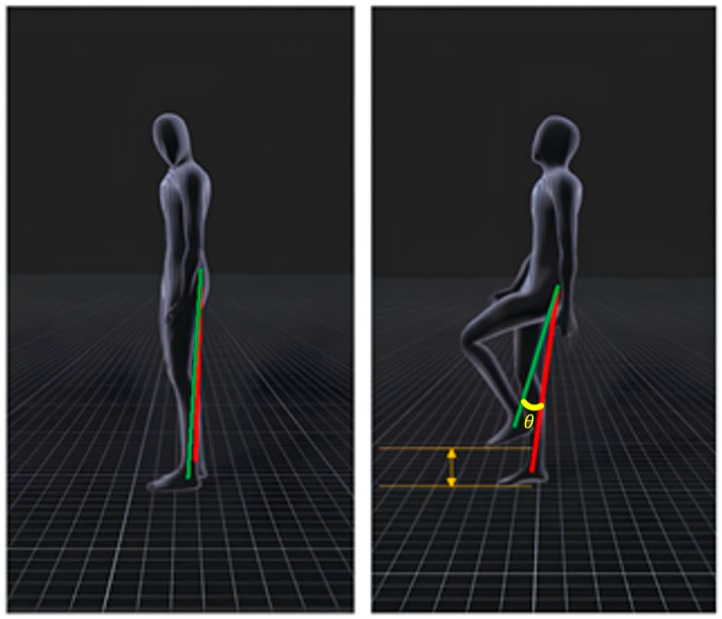
Implementation of the proposed WIP technique with Mocopi.

Various physical movements can be observed while a person walks, such as arms swinging, knees bending, and the soles of the feet lifting off the ground. This study proposes an algorithm based on the behavior of a user walking in place to analyze body movements during walking using data from sensors attached to the ankle and waist. It involves the user walking in place, with one foot on the ground and the other off the ground. The imaginary line formed by the foot on the ground and the waist sensor is called “baseline *A*,” whereas that formed by the foot off the ground and the waist sensor is called “baseline *B*.” The angle between the two reference lines is measured to determine the user movement, as illustrated in Figure 3. The angle formed by the two baselines is calculated as follows:

(1) $$cos\theta =\frac{A\cdot B}{\left\|A\right\|\left\|B\right\|},$$

(2) $$\theta ={cos}^{-1}\left(\frac{A\cdot B}{\left\|A\right\|\left\|B\right\|}\right),$$

where (1) represents the dot product, and (2) represents the transformation of (1).

**Figure 4:**
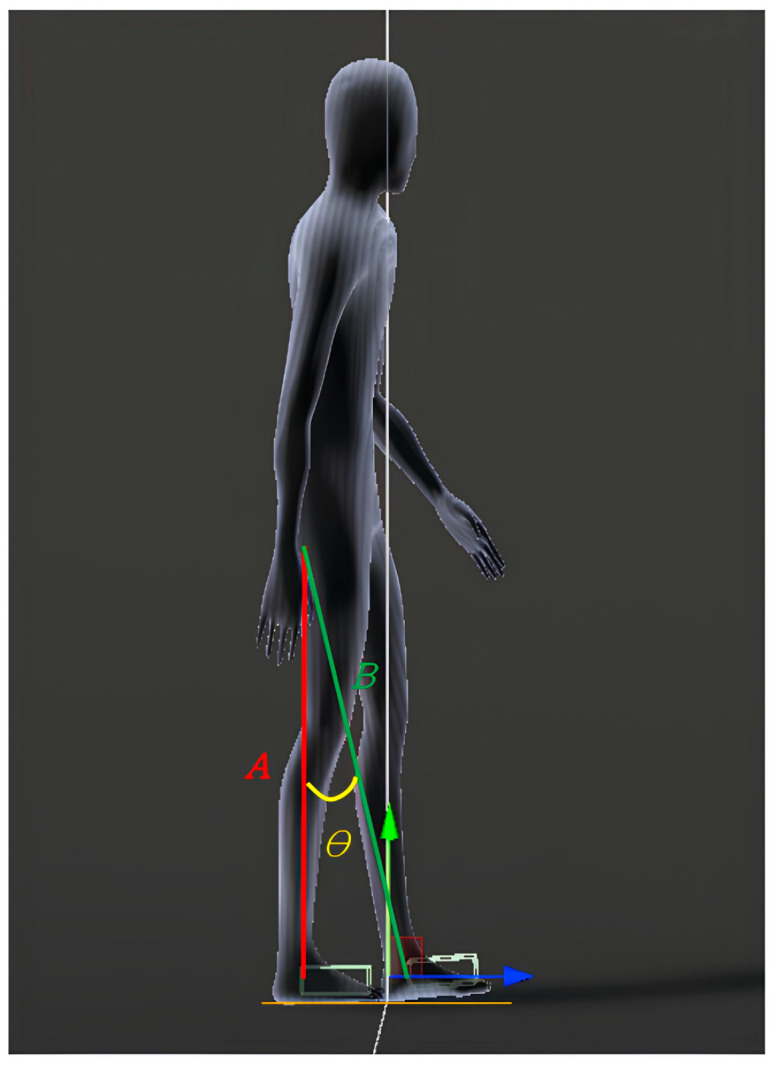
Exceptions to applying WIP.

**Figure 5:**
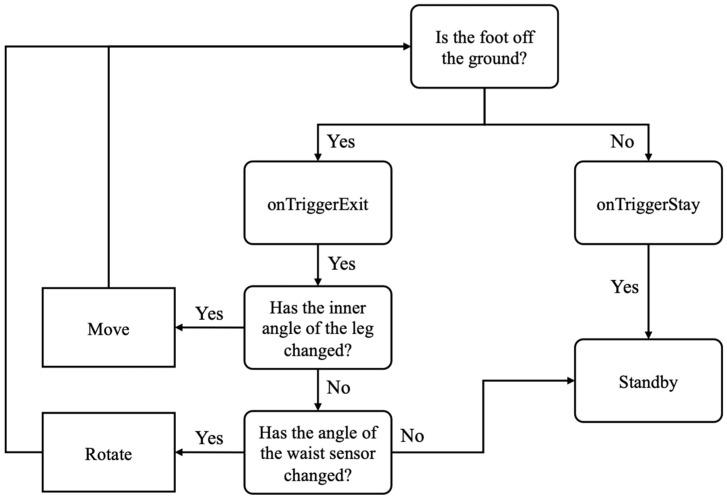
WIP implementation process.

Although the angle between the two baselines was used to determine the gait of the user, using this method alone can cause malfunctions in certain situations. For instance, the angle change that occurs while the user is standing, as shown in Figure 4, can be mistaken for a walking motion. This confusion can arise when the user is performing a WIP motion or raising and lowering their legs. To address these issues, this study presents a technique for detecting the moment when a foot touches the ground and falls by attaching a collider component to the 3D model. A collider is a physical collision-detection component in the Unity game engine that precisely detects the interactions between the foot and the ground. The “OnTriggerStay” function is activated when the foot comes into contact with the ground, whereas the “OnTriggerExit” function is activated when it falls. In Unreal engine, we can use “OnComponentBeginOverlap” and “OnComponentEndOverlap” functions. This allowed us to precisely determine the user’s movement based solely on the internal angle when the foot is not in contact with the ground. Figure 5 shows the process of implementing WIP using the aforementioned internal angles.

### 3.9. Designing the WIP User Interface

**Figure 6:**
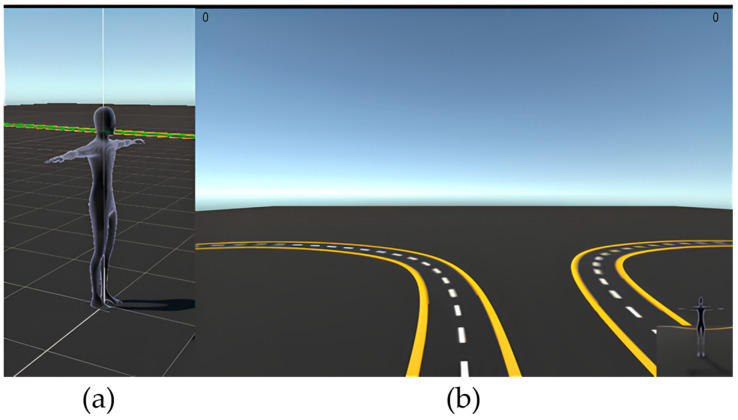
(**a**) Three-dimensional model based on captured motion data and (**b**) view of the user wearing the HMD, including their orientation and movement information.

**Figure 7:**
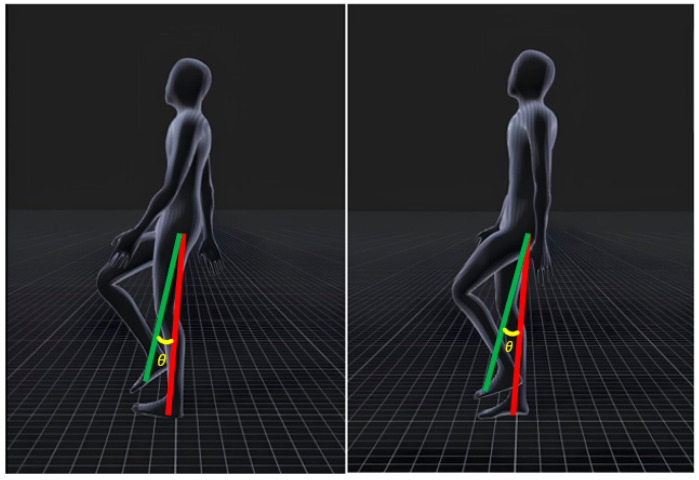
Walking motion for right and left foot.

A visualization method was employed to display the captured motion data to users. Figure 6a shows a 3D Unity model of the user’s body data collected via Mocopi, whereas the user’s point of view while wearing the HMD and experiencing it is shown on Figure 6b. Figure 7 shows how to visually verify the walking motion, and the angles of the right and left feet. That is shown in Figure 6b as text on the top left and right corners of the screen, respectively. A sub-camera was placed at the bottom right to track the entire body and verify the direction of movement. The white dotted line at the center of the road was generated based on the target data. The width of the road, marked with a yellow solid line, was set to 1 m.

## 4. Experiments

### 4.1. Experimental Setup

**Figure 8:**
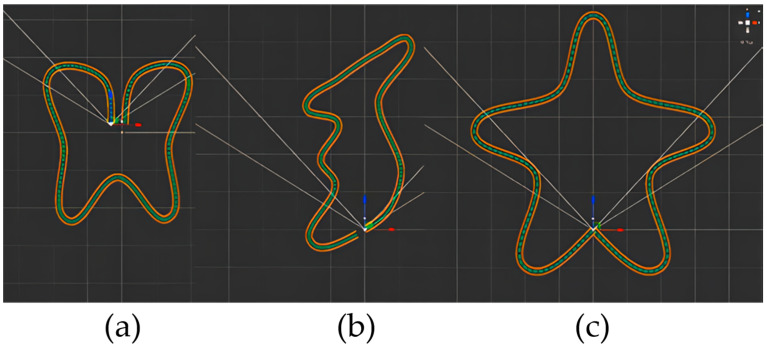
Three target paths: (**a**) butterfly-shaped, (**b**) Korean Peninsula-shaped, and (**c**) star-shaped.

The experiment used the Vector3 values generated by traveling along the three pre-created paths shown in Figure 8 to evaluate the performance of the proposed WIP technique with Mocopi. The 1 m wide path was created using the Bézier Path Creator asset, which is a free asset. Comparative data were extracted through comparisons with a treadmill, which is a device, and the results are presented through graphs and values. The experiment included 16 participants of both sexes with varying ages, ranging from 20 to 70. The participants were divided into two groups of eight each, using a between-subjects design comprising Mocopi and treadmill conditions. To ensure that the participants did not gain knowledge of the maps and the WIP technique during the experiment, they experienced either the Mocopi or treadmill, and the maps were presented in a random order. Furthermore, an objective survey was employed to evaluate both methods. The experiment was conducted under the following conditions:

- Application: Unity 2021.3.8.f1.
- System specifications: AMD Ryzen 5 5600×/32 GB RAM/AMD RX 6700 XT GPU with 12 GB of GDDR6 memory.
- HMDs: Meta Quest2 and VIVE Pro.
- Trackers: Mocopi and KAT Walk Mini S.

To obtain the Vector3 values of the map, the position values were extracted point-by-point in the CSV format for the *x*-, *y*-, and *z*-axes, with the *y*-axis set to zero to align it with the ground. Table 2 summarizes the number and size of the Vector3 values of the target path.

**Figure 9:**
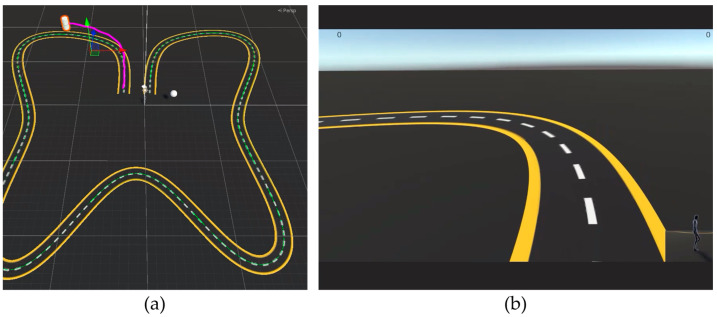
User’s (**a**) path and (**b**) viewpoints.

The line renderer feature was used to visualize the user’s path and extract the collected data in the CSV format, as shown in Figure 9. Only the *x* and *z* values were required for data comparison; therefore, the *y* value was set to zero.

### 4.2. Experimental Results

#### 4.2.1. Performance Evaluations

This study employed ATE, a commonly used metric for path evaluations, using data obtained from the target and actual path values of the user. The ATE is an important metric in various applications and is primarily related to path tracking. It measures the error between the target and user paths, wherein a smaller value indicates higher matching between the paths.

The user index must remain constant to obtain an accurate cumulative distance ATE. However, the obtained Vector3 index is always different because not all users travel the same path. In this study, new points were created after every 1 m based on the distance of the user path to obtain accurate ATE values. Additionally, new points were created for the target path at 1 m increments for the same environment, and the absolute path lengths are listed in Table 3.

Sixteen users participated in the experiment, consisting of a variety of ages ranging from their 20s to 60s, and included both genders. To ensure a fair trial, the participants were split into two groups: eight users experienced the Mocopi and the other eight used the treadmill.

**Figure 10:**
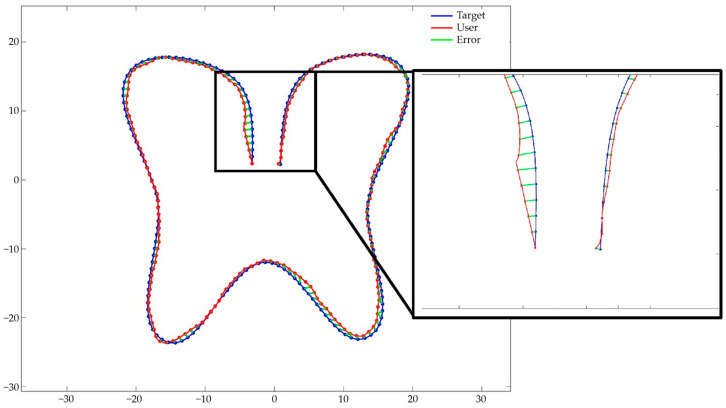
Plotted path and error.

**Figure 11:**
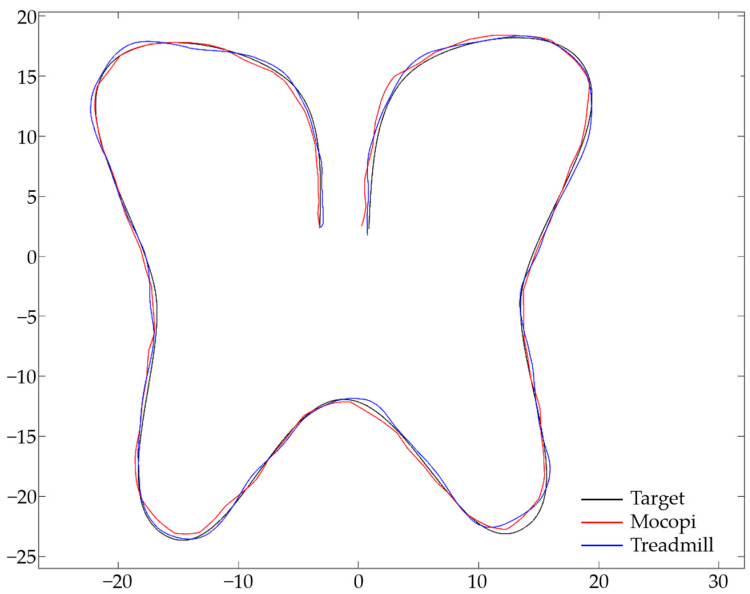
Mocopi, treadmill, and target paths for a user who experienced the butterfly-shaped path.

**Figure 12:**
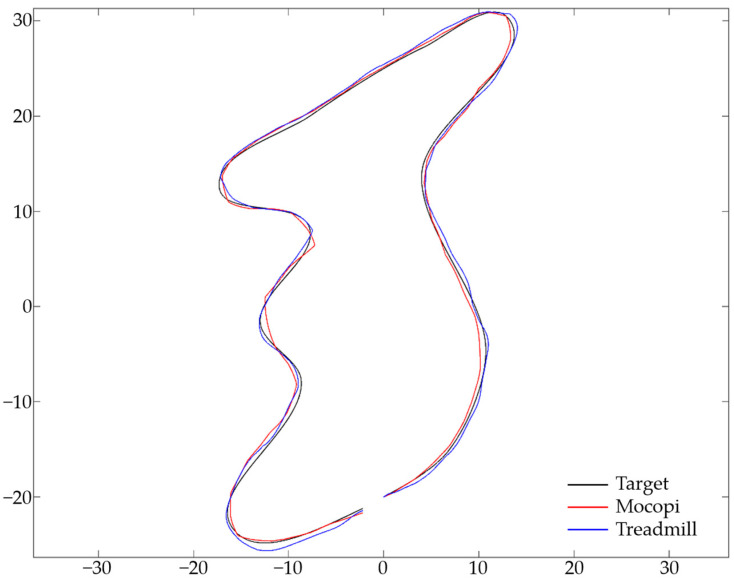
Mocopi, treadmill, and target paths for a user who experienced the Korean Peninsula-shaped path.

**Figure 13:**
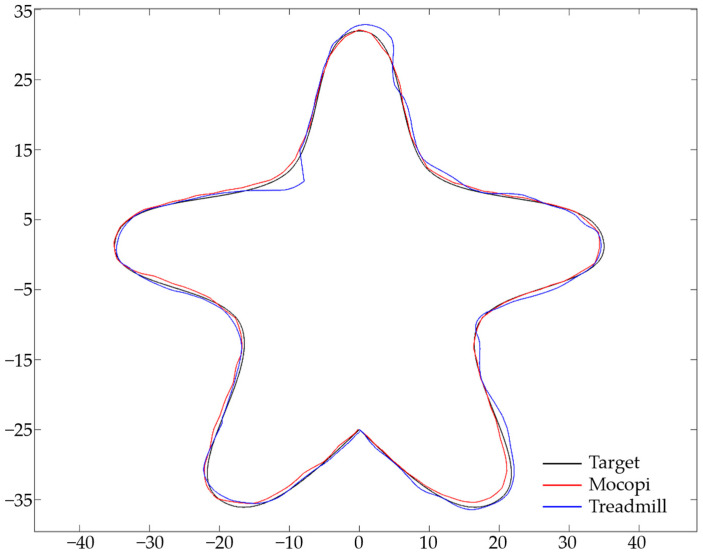
Mocopi, treadmill, and target paths for a user who experienced the star-shaped path.

To obtain the ATE values, the target and user path data were matched in a 1:1 ratio, and ATE (m) was calculated by measuring and adding the distances between points. Figure 10 illustrates the target (blue) and user (red) paths with each point matched. The difference in the distance between the target and user paths is indicated by the green line. Figure 11 illustrates the Mocopi, treadmill, and target paths for one of the users who experienced the butterfly-shaped path, and Table 4 presents their ATEs. Comparisons with the corresponding ATE values show that Mocopi was more accurate, with a mean ATE of 61.48 compared to 66.30 for the treadmill. However, the standard deviation between the treadmill users is better. Figure 12 shows the Mocopi, treadmill, and target paths for one of the users who experienced the Korean Peninsula-shaped path, and Table 5 presents their ATEs. The average ATE of the treadmill users was 64.48, whereas that of Mocopi users was 63.59, indicating that Mocopi outperformed the treadmill. However, the standard deviation between the treadmill users is better. Figure 13 illustrates the Mocopi, treadmill, and target paths for one of the users who experienced the star-shaped path, and Table 6 lists their ATEs. The average ATE of the treadmill was 64.48 points, whereas that of Mocopi was 63.59 points, indicating that Mocopi outperformed the treadmill; however, the standard deviation between treadmill users was better.

By comparing the WIP performances of the Mocopi and treadmill users with the ATE results, slight variations were observed among individuals; however, they were insufficient to be noticeable in VR. However, the graph and the tables indicate that some users recorded higher than average ATE values. We investigated the causes for this through interviews, and found that this was primarily caused by inexperience. Reasons other than poor treadmill manipulation were identified as unwanted movements caused by the sensor reacting to objects other than the feet, such as clothing, resulting in mis-operation.

#### 4.2.2. User Evaluations

Although ATE figures provide statistical representations of the target and user paths, they do not include several evaluation metrics such as user immersion, wearability, and spatiality. This study comprehensively evaluated the user experiences of using the proposed WIP in conjunction with Mocopi and a treadmill through a survey comprising nine questions regarding immersion, wearability, convenient, reality, difficulty, responsiveness, connectivity, freedom, and spatiality, as follows:

- Immersion: Is the level of immersion in your WIP experience satisfactory?
- Wearability: Did you experience any physical discomfort during your WIP experience?
- Convenience: Were you physically comfortable during your WIP experience?
- Reality: Are you satisfied with the realism of WIP?
- Difficulty: What were some of the challenges you encountered while trying out WIP?
- Responsiveness: Are you satisfied with the real-time movement of WIP?
- Connectivity: How would you evaluate the connection between the physical and virtual worlds when using WIP technology?
- Freedom: What is your evaluation of the increased freedom provided by WIP?
- Spatiality: Are you satisfied with the space allocated in WIP?

Each question could be answered with a rating on a scale of 1–5, and a higher score indicated a better evaluation. The survey was administered to all 16 participants.

Among the nine survey questions, as shown in Table 7, Mocopi scored higher than the treadmill in four. The treadmill scored higher for immersion, realism, responsiveness, and connectedness because it employed a normal walking motion on a slip pad rather than just walking in place. However, the treadmill scored lower than Mocopi for wearability, difficulty of use, ease of use, and space because it requires considerable energy to operate and is bulky, making it difficult to move it from one place to another.

## 5. Discussion

### 5.1. Limitations

The low-cost motion-capture device employed in this study, Mocopi, exhibits several advantages. However, it is important to note that it also presents several technical limitations. One such limitation is the potential for delays to occur in the real-time processing and transmission of sensor data. This is largely due to the inherent limitations of data transmission over Bluetooth connectivity. Such delays can potentially lead to a reduction in the immersion and user experience of VR, which is a significant factor in this regard. This represents a significant challenge, particularly for VR applications that necessitate dynamic and real-time responses. Consequently, future research should focus on technical improvements to minimize this delay. Furthermore, the development of more reliable and faster data transfer protocols is essential to address connectivity issues. These technological advances will contribute significantly to the commercialization of VR technology and improve the quality of the user experience.

One of the limitations of this study is the small sample size. The dataset used is not large enough to allow for generalizability of the results. In particular, a larger and more diverse sample is needed to assess the effectiveness of the algorithm in different settings and conditions. This could be an important factor in assessing the universality and reliability of the algorithm, and future research should be conducted using a larger sample.

### 5.2. Theoretical Implications 

This research extends existing theories on VR technology through the development of a low-cost VR tracking algorithm. In particular, it is significant in that it advances our understanding of how to make VR technology more cost-effective and accessible. By exploring the impact of low-cost technologies on the performance and user experience of VR systems, this research strengthens the theoretical foundations of technology acceptance models and user experience. Furthermore, it contributes to the theoretical debate on the impact of technological innovation on user adoption by presenting different approaches to lowering the barriers to VR technology through efficient cost structures.

### 5.3. Managerial Implications

The results of this study have significant implications for companies that are considering the commercial exploitation of VR technology. The development of low-cost algorithms provides an opportunity to make VR technology accessible, especially to small and medium-sized enterprises and startups with limited budgets. This will enable companies that are interested in utilizing VR in education, training, marketing, product development, and other areas to deliver high-quality VR experiences at a lower cost. The research also provides useful guidance for the development of marketing strategies to promote the adoption of VR technology and reach a wider range of users. Companies can use the findings to develop more affordable VR solutions and create strategies to increase user acceptance of the technology.

### 5.4. Significance of the Results 

The low-cost VR tracking algorithm developed in this research represents a significant step forward in lowering the cost barrier for VR technology and making it accessible to a wider range of users. By demonstrating that efficient tracking can be achieved at low cost, it opens the door to a wider range of applications for VR technology in education, training, and entertainment. This study also provides empirical evidence of the positive impact that the development of a low-cost tracking solution has on the user’s VR experience. These data are of significant value for theoretical discussions on technology acceptance models and user experience. Future research can further develop these techniques and contribute to the commercial utilization and widespread adoption of VR technology.

## 6. Conclusions

VR technology has significantly advanced over the years. Although improvements in hardware performance have enhanced its visual impact, providing realistic and natural interactions between the user and VR environment remains challenging. We aimed to allow users to move freely between the real and virtual environments, thereby providing an excellent experience without physical constraints.

This study employed Mocopi, a low-cost motion-capture device, to collect and analyze user movements in real time. Mocopi comprises six small sensors that must be attached to the user’s body and can operate without external sensors. The proposed WIP algorithm can control user movements in a VR environment more naturally without spatial constraints.

To compare the performance of the WIP algorithm, the participants were asked to walk along three target paths using an expensive treadmill, and their ATE values were compared. The results showed that Mocopi performed better than the treadmill. To further evaluate the user experience, we conducted a user survey and found that the treadmill was superior in terms of immersion and realism, whereas Mocopi was superior in terms of spatiality, convenience, and wearability. These results demonstrate the superiority of the proposed WIP algorithm when using Mocopi in VR environments.

In this study, we attempted to mitigate the limitations of the sample size by employing multiple paths, including a star, butterfly, and peninsula shapes. Despite these efforts, we recognize that the small sample size may affect the results of the study. In future studies, we will strive to overcome this limitation by increasing the sample size.

This study strived to make immersive VR experiences more accessible by introducing a low-cost, real-time, full-track technology that can reduce costs by at least 3 times and up to 20 times compared with other WIP techniques. Thus, it can allow users to have an excellent VR experience and break the boundaries between real and virtual environments without incurring excessive costs. As a potential avenue for future research, it is necessary to investigate the possibility of seamlessly experiencing real-world movements in a VR environment without additional motion recognition devices and controllers.

## Figures and Tables

**Table 1:** Indexes and names of joints defined in Mocopi.

Index	Joint Name	Index	Joint Name
0	root	14	left_hand
1	torso_1	15	right_shoulder
2	torso_2	16	right_up_arm
3	torso_3	17	right_low_arm
4	torso_4	18	right_hand
5	torso_5	19	left_up_leg
6	torso_6	20	left_low_leg
7	torso_7	21	left_foot
8	neck_1	22	left_toes
9	neck_2	23	right_up_leg
10	head	24	right_low_leg
11	left_shoulder	25	right_foot
12	left_up_arm	26	right_toes
13	left_low_arm		

**Table 2:** Target path extraction data.

Path	Butterfly	Korean Peninsula	Star
CSV Vector3 index	2681	2392	2067
Map size	44 × 43 m	34 × 58 m	72 × 70 m

**Table 3:** Target path total distance (m).

Path	Butterfly	Korean Peninsula	Star
Path distance	180	160	250

**Table 4:** ATEs for the butterfly-shaped path.

Mocopi		Treadmill	
User	ATE	User	ATE
user1	50.24	user1	76.66
user2	63.73	user2	70.79
user3	61.84	user3	68.05
user4	78.35	user4	69.99
user5	57.01	user5	56.10
user6	53.75	user6	65.81
user7	67.22	user7	69.16
user8	59.66	user8	53.81
Average	61.48	Average	66.30
Standard deviation	8.16	Standard deviation	7.18

**Table 5:** ATEs for the Korean Peninsula-shaped path.

Mocopi		Treadmill	
User	ATE	User	ATE
user1	98.38	user1	49.83
user2	55.81	user2	70.58
user3	70.24	user3	53.18
user4	56.15	user4	67.97
user5	61.45	user5	58.91
user6	50.11	user6	86.61
user7	67.84	user7	61.78
user8	48.71	user8	66.94
Average	63.59	Average	64.48
Standard deviation	14.98	Standard deviation	10.76

**Table 6:** ATEs for the star-shaped path.

Mocopi		Treadmill	
User	ATE	User	ATE
user1	117.85	user1	70.45
user2	102.85	user2	105.00
user3	84.02	user3	112.73
user4	96.10	user4	126.35
user5	80.26	user5	104.38
user6	110.11	user6	99.64
user7	79.94	user7	100.01
user8	74.48	user8	75.48
Average	93.20	Average	99.26
Standard deviation	14.89	Standard deviation	17.21

**Table 7:** Survey results.

Evaluation Elements	Mocopi		Treadmill	
	Average	Std. dev	Average	Std. dev
Immersion	4.375	0.484	4.625	0.484
Wearability	4.625	0.695	4	0.707
Convenience	4.5	0.5	3.625	0.695
Reality	4.125	0.78	4.5	0.707
Difficulty	4.375	0.484	4.125	0.599
Responsiveness	4.25	0.433	4.625	0.484
Connectivity	3.875	0.599	4.5	0.5
freedom	3.875	0.599	4	0.5
Spatiality	4.75	0.433	3.375	0.484

## Data Availability

The raw data supporting the conclusions of this article will be made available by the authors on request.
